# Assessment of the burden of rabies in one health approach control program in Ketapang District Indonesia: Using zDALY

**DOI:** 10.1016/j.pmedr.2024.102838

**Published:** 2024-07-26

**Authors:** Cut Desna Aptriana, Etih Sudarnika, Chaerul Basri, Dikky Indrawan, Joko Daryono, Pebi Purwo Suseno

**Affiliations:** a Directorate of Veterinary Public Health, Ministry of Agriculture Republic Indonesia, Jakarta 12550, Indonesia; b School of Veterinary Medicine and Biomedical Science, IPB University, Bogor 16680, Indonesia; c School of Business, IPB University, Bogor 16153, Indonesia; d Australia Indonesia Health Security Partnership (AIHSP), Jakarta 12550, Indonesia; e Directorate of Animal Health, Ministry of Agriculture Republic Indonesia, Jakarta 12550, Indonesia

**Keywords:** Animal loss equivalent, DALY, Disease burden, One health, Rabies, zDALY, Zoonoses

## Abstract

**Background:**

Ketapang District, located in West Kalimantan, is a region where rabies is endemic. The first human death from rabies was reported in 2014 and the problem persists to this day. In response, the government has implemented the one health approach to control rabies since 2017. This study aimed to assess the disease burden of rabies control using zDALY metrics.

**Methods:**

The zoonotic burden for human and animal was measured by developing Zoonotic Disability-Adjusted Life Years (zDALY), which combines DALY and local values of animals and their products, considering animal morbidity and mortality due to the disease (Animal Loss Equivalent/ALE). Data were gathered through interviews with victims or their families, dog owners, and secondary data from the Animal Husbandry Service, and the District Health Office.

**Results:**

Before implementing the one health approach, the zDALY value was 1,561.22 person-years (the zDALY rate: 109.53 years/100,000 people). After the intervention, the zDALY value was 1,808.86 person-years (the zDALY rate: 86.62 years/100,000 people).

**Conclusions:**

This reduction in the zDaly rate indicates that rabies control through the one health approach in Ketapang District was effective in alleviating the disease burden caused by rabies.

## 1 Introduction

Rabies posing a continued global threat with significant social and economic consequences (WOAH, 2021). In East Nusa Tenggara, Indonesia, the economic impact from 1998 to 2007 was estimated at 14.2 billion rupiahs per year. The economic impact was distributed across various components, with the highest cost attributed to culling roaming dogs, accounting for 39 % of the total cost. Other components included post-exposure treatment (PET) (35 %), mass vaccination (24 %), pre-exposure treatment (1.4 %), and others (1.3 %) (dog bite investigation, diagnostic of suspected rabid dogs, traceback investigation of human contact with rabid dogs, and quarantine of imported dogs) (Wera et al., 2013).

According to the WHO (2018), rabies causes 59,000 human deaths annually, about 95 %, occur in Africa, and Asia and are particularly prevalent in rural poor communities. So the prevention and control of rabies are crucial, requiring significant investment, resource allocation, and commitment from various sectors to participate in the control program. The rabies control program employing the one health approach is considered effective because it involves cross-sector collaboration between animal and human health sectors through to enhance research and surveillance concerning zoonoses and emerging diseases (Directorate General of Livestock and Animal Health of Indonesia, 2019, Balia et al., 2019).

The assessment of disease burden involves quantifying the cost, time, and energy lost due to health-related condition. Disease burden encompasses the cumulative impact of medical, economic and psychosocial costs associated with a specific disease condition (Aikins and Agyemang, 2016). Generally, the measurement of disease burden utilizes disability-adjusted life years (DALY), which combines the years of life lost (YLL) due to premature death and the years lost due to disability (YLD) as years lived with disability/disease. For zoonotic diseases, the burden of disease extends beyond the impact on humans to include economic losses in animals (animal loss equivalent/ALE). As a result, the burden of zoonotic diseases is measured using zoonotic disability-adjusted life years (zDALY), which considers the YLL, YLD, and ALE. Specifically, the measurement of rabies burden is estimated through zDALY.

West Kalimantan is one of the provinces in Indonesia that has been affected by rabies. Initially, the government attempted to control rabies in Ketapang District from 2014 to 2016 without employing the one health approach. During this period, the control activities lacked cross-sectoral collaboration, leading to suboptimal reporting of bite cases and inadequate case records. However, from 2017 to 2020, in collaboration with the Food and Agriculture Organization, the Ministry of Agriculture initiated a control program using the one health approach. The intervention involved advocating for and training officers in both the animal health and human health sectors on the application of the one health approach. The training proved instrumental in fostering a foundation for the implementation of one health in the Ketapang District, with improved coordination, communication, and collaboration. The positive impact of the one health approach became evident in the more effective and manageable vaccination activities in animals, as well as improved information, education, and communication efforts, which could now reach a wider audience.

Considering the absence of rabies burden measurement studies in Ketapang District West Kalimantan, the objective of this study is to estimate the rabies burden disease using zDALY measurement. This study aims to provide additional information to decision-makers in Ketapang District and the West Kalimantan Province Government, emphasizing the significance of implementing a rabies control program.

## 2 Materials and methods

### 2.1 Ethical approval

This study received ethical clearance and informed consent approval from the Human Research Ethics Committee of IPB University under reference number 695/IT3.KEPMSM-IPB/SK/2022.

### 2.2 Study design and study population

This study used morbidity and mortality data from the Department of Animal Husbandry and Animal Health in West Kalimantan Province and Ketapang District, as well as data from West Kalimantan Provincial Health Service and Ketapang District. Additionally, in-depth interviews were conducted with dog owners and patients who had bitten by dogs in Ketapang District. A total of 150 respondents were included in the interviews, with 50 respondents from each of the following sub-districts: Tumbang Titi, Delta Pawan, and Sungai Melayu Raya. These sub-districts were selected due to their high incidence of dog bites on humans. The selection criteria for respondents were individuals who owned dogs and those who had experienced dog bites. The interviews were carried out in the three sub-districts by three trained enumerators. A structured questionnaire was used during the interviews. Each sub-district had 25 dog owners and 25 individuals who had been bitten by a dog, totaling 50 respondents per sub-district. For clarification, individuals who had been bitten by a dog but the dog was not infected with rabies were considered as respondents for the study. The respondents were asked about cases that occurred within the 2021 period, covering one year. The interview questions for dog owners covered personal data of the respondents (gender, age, occupation, address, income, education), information about their animal ownership (purpose of animal, type of animal, number of animals owned, and total maintenance costs). On the other hand, the interview questions for individuals who had been bitten by a dog included personal data of the respondents (gender, age, occupation, address, income, education), details about the bite incidents they experienced (time, location, and actions taken after the bite), distance from the incident location to the health facility, the duration of treatment after the bite, and the associated treatment costs. All the cases referred to in the interviews occurred within a one-year period in 2021.

The duration and disability weight of rabies disease were obtained through interviews, and the calculation of the Disability-Adjusted Life Years (DALY) was done using the DALY calculator (Global Health CEA, 2022). The life expectancy data for the population in Ketapang District in 2020 was obtained from the Central Bureau of Statistics. Data collection took place from January 2021 to April 2022, covering both Pontianak City, and Ketapang District in West Kalimantan Province. Trained interviewers conducted face-to-face interviews during the data collection process. Afterward, the collected data was processed at the Epidemiology Laboratory of the School of Veterinary Medicine and Biomedics, Bogor Agricultural University.

### 2.3 Data analysis

This study assessed the burden of zoonoses with zDALY. The calculation of DALY for zoonoses (zDALY) is by adding YLL, YLD and the estimated ALE value from the monetary value of livestock losses and per capita local income. The calculation of YLD and YLL was obtained using the DALY calculator on https://ghcearegistry.org/orchard/daly-calculator (Global Health CEA, 2022). The input in the calculation of the DALY calculator was the value obtained through a questionnaire survey such as the average age of onset; the average age of death; the incident cases; and the incident death. In this DALY estimation, we did not distinguish gender so the calculation applies to both males and females. YLD is determined based on the years lost due to disability experienced by individuals affected by certain zoonotic diseases. To calculate YLD, the number of dog bite cases (I), the duration of the disease with zoonotic consequences (L), and the disability weight (DW) representing the severity of the disease on a scale from 0 to 1 (where zero indicates perfect health and one indicates death) are multiplied together. The estimation of DW requires information about the level of morbidity, while the duration (L) is essential for calculating YLD (Devleesschauwer et al., 2014). DW of 0.133 was obtain from Global Health CEA. YLL was calculated by multiplying the number of deaths (N) with the remaining life expectancy (L) of the population in the Ketapang District.

YLD=I×L×DW;YLL=N×L

DALY=YLD+YLL

Information regarding the components of ALE was collected from morbidity and mortality data related to livestock and other domestic animals considering the local values and their products. The ALE data in this study was obtained through a questionnaire administered to animal owners, which provided valuable information about the animal's worth. This included factors such as (1) the roles of dogs (e.g., gardeners, hunters, and pets), (2) the number of hours the dogs worked, and (3) the conversion of the wages earned if the same tasks were performed by humans. This allowed the researchers to ascertain the monetary value of the animals. As rabies cases in Ketapang District, West Kalimantan Province, were limited to being reported in dogs, the ALE calculation focused on the function value of dogs in that specific area. The total monetary impact of the disease on this animal was then divided by the Gross National per capita Income (GNI) for the year 2020 to obtain ALE value.

ALE=monetary value of animal health losses/GNI per person

All calculations were conducted using nominal values, converted to Rupiah (IDR) based on the average exchange rate in 2020, as other studies also utilize current local currency values. The calculation of zDALY combines DALY and ALE values. The estimation of ALE was based on this conversion. The zDALY in this study was calculated before and after the intervention with the one health approach.

zDALY=YLL+YLD+ALE

GNI per capita data in the current dollar, according to World Bank figures (downloaded from: https://data.worldbank.org/indicator/NY.GDP.MKTP.CD?locations=ID) for reference in the study.

## 3 Results

### 3.1 Demography and rabies history

**Fig. 1:**
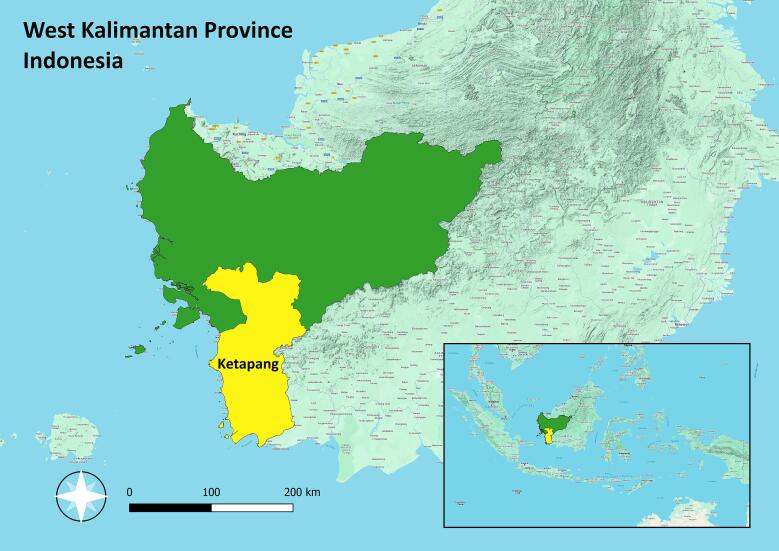
West Kalimantan Province Map (Source: Map generated by QGIS 3.22.5 software).

Ketapang District, the largest district in West Kalimantan Province (Fig. 1), spans 31,588 km^2^, a land area of 30,099 km^2^ and a water area of 1,489 km^2^. This district focuses on agricultural, plantation, mining, forestry, fisheries, industrial and tourism sectors (Audit Board of Indonesia, 2023). In Ketapang District, dogs serve as guardians for gardens. The prevailing system of dog maintenance in the area involves releasing the dogs instead of keeping them in cages. However, the vast expanse, substantial human resources, budget constraints, and limited animal health service facilities present significant challenges for the government in controlling rabies in this region.

Since 1974, rabies has been endemic in Kalimantan, with the first case in East Kalimantan. Furthermore, Central Kalimantan reported three cases in 1978, South Kalimantan in 1983, and West Kalimantan in 2005. However, West Kalimantan successfully eliminated rabies in August 2014 (Umboh et al., 2018). Unfortunately, in October 2014, the first rabies case in Ketapang District emerged, leading West Kalimantan to the list of areas affected by rabies, with 13 out of 14 districts are infected.

### 3.2 Analysis of rabies burden in Ketapang District

Before the implementation of the one health approach from 2014 to 2016, Ketapang District recorded 429 cases of dog bites on humans and 13 cases of human deaths caused by rabies. Since the case fatality rate for rabies in humans is nearly 100 %, and the average age of exposure to rabies is 15 years, considering a life expectancy of 72.67 years in Ketapang District (Central Bureau of Statistics Indonesia, 2021), the reported disease duration with zoonotic symptoms is 14 days, the disability weight of 0.133, a YLD of 799 was obtained. Additionally, a YLL of 762 and a total DALY of 1,560.99 person-years were calculated or monetized with Gross Domestic Product (GDP) reaching 84.46 billion Rupiah.

In Ketapang District, the reported cases involved only dog bites, no other animals were suspected rabies. Before of the one health approach in the rabies control program, the calculated ALE calculation was 0.23. The zDALY value the implementation of the one health approach was 1,561.22 per person-years or 109.53 per 100,000 person-years. However, after the intervention with the one health approach, there was an increase in the number of reported dog bite cases on humans, rising from 429 to 877 cases. This increase was attributed to better reporting practices, leading to a reduction in under-reporting cases. On a positive note, the number of human deaths due to rabies decreased from 13 to 3 individuals. Disease duration and disability weight obtained the same valuable information as before the one health intervention. The YLL value obtained from this situation was 176, while YLD was 1,633, resulting in a total DALY of 1,808.86 person-years or monetized with Gross Domestic Product (GDP) reaching 97.87 billion Rupiah.

Following the implementation of the one health approach in the rabies control program in Ketapang District, the ALE value increased to 0.48. The zDALY value after the one health approach was introduced reached 1,809.35 person-years or 86.82 per 100,000 person-years. Further information on the components and calculation details of the zDALY value before and after the one health approach in the rabies control program from 2014 to 2016 in Ketapang District can be found in Table 1.

**Table 1:** The zDALY component calculation before and after the intervention with the one health approach to the rabies control program in Ketapang District.

**Component**	**Code**	**Formula**	**Result**		**Reference**
			**Before the intervention with one health approach**	**After the intervention with one health approach**	
- Total rabies case	I	Number of bite cases of rabies transmitting animals to humans/animals	429	877	Plantation and Livestock Service of West Kalimantan Province, 2022
- Animal Value	NH	Animal value according to animal function (in Rupiah)	30.000	30.000	Plantation and Livestock Service of West Kalimantan Province, 2022
- *Gross Domestic Product*	GDP	The share of Indonesia's national income with the total population of Indonesia (in Rupiah)	54.110.680	54.110.680	The World Bank, 2022
- Death cases	N	Total death cases caused by rabies	13	3	West Kalimantan Provincial Health Office, 2022
- Life Expectancy	L	(in years)	72,67	72,67	Central Bureau of Statistics Indonesia, 2021
- Life expectancy at age of premature death	L1	(in years)	58.67	58.67	Global Health CEA, 2022, Central Bureau of Statistics Indonesia, 2021
- The disease duration with zoonotic symptoms	L2	(in days)	14	14	Global Health CEA, 2022
- Disability weight	DW		0,133	0,133	Global Health CEA, 2022
- *Animal loss equivalents* (ALE) *averted*	ALE	ALE = (total rabies cases (I) × NH)/GDP	0,23	0,48	Plantation and Livestock Service of West Kalimantan Province, 2022
- *Years Life Lost*	YLL	YLL = total deaths (N) × Life expectancy at age of premature death (L1)	762	176	West Kalimantan Provincial Health Office, 2022, Central Bureau of Statistics Indonesia, 2021
- *Years Life with Disability*	YLD	YLD = total cases (I) × remaining life expectancy with zoonotic symptoms (L2) × disability weight (DW)	799	1.633	West Kalimantan Provincial Health Office, 2022, Global Health CEA, 2022
- DALY	DALY		1.560,99 year-person	1.808,86 year-person	
- Zoonosis DALY	zDALY	DALY+ALE	1.561,22 year-person	1.809,35 year-person	
- *Rate* zDALY	*Rate* zDALY		109,53 year per 100.000 population	86,82 year per 100.000 population	

## 4 Discussion

Ketapang District is a rabies endemic area so that provincial government policy considers all suspected dog bite cases as rabies cases in humans so that all dog bite cases that occur were carried out PEP treatment. Each case of dog bite occurs assessed 1 (one) dog bites 1 (one) human person, so this assessment will certainly affect the calculation of ALE.

The zDALY framework addresses the gap in assessing the disease burden arising from the transmission between animals and humans (Shaw et al., 2017). Comparing zDALY rates, it was found that the rate before the implementation of one health approach was higher than after the intervention. It implies controlling zoonoses necessitates a one health approach. The estimation of the zDALY value in this study utilizes a deterministic model, which means that the confidence interval for the estimated value is not calculated. This can be considered a limitation of the study.

The one health approach increased the DALY value. The prompt response and improved reporting dog bite cases contributed to the decline in human deaths due to rabies. The effectiveness of the one health implementation in Ketapang District is evident in reduction of rabies cases. However, program sustainability and enhancement requires strong commitment and support from local governments, including legal frameworks and adequate budget allocations to enhance the performance of field officers (Aptriana et al., 2022). The presence of unreported dog bite cases and human deaths due to rabies can influence the assessment of the disease burden. Limited access to health facilities, difficulties in reporting cases, and individuals opting for self-medication after being bitten contribute to under-reporting. These factors have led to a decrease in the burden rate of this disease following the intervention with the one health approach.

Globally approximately 3,700,000 DALYs are estimated to be lost due to rabies, with a total DALY of 12,311 reported for Indonesia (Hampson et al., 2015). The YLL was based on the number of reported human rabies clinical cases, considering the breakdown by age. This study utilized a disability weight of 0.133 (Global Health CEA, 2022) with an average duration of 14 days to calculate the YLD for patients who suffered from bite wounds and underwent post-exposure treatment. In contrast to the scenario presented by Sultanov et al. (2016) used a different disability weight of 0.108 and an average duration of 60 days to estimate YLD. It should be noted that the disability weights differ depending on factors such as the quality of life adjusted for years of life109.53, health status assessment, and health status preferences (Minsu et al., 2019).

In Colombo, Sri Lanka, the zDALY value is recorded as 740 (Häsler et al., 2014). This value is lower compared to the zDALY in Ketapang District, primarily because the calculation of zDALY in Colombo assumes the value of ALE as zero, indicating that dogs are considered to have no economic value in that context. In a systematic review study focusing on the burden of zoonoses in Paraguay conducted by Zayas et al. (2021), zDALY value for rabies in Paraguay was found to be 159. This value was lower than the zDALY in Ketapang District due to the absence of included human death cases o caused by rabies in the collected studies from 2000 to 2019. The reported death cases were primarily observed in dogs and livestock, resulting the zDALY calculation considering only the burden of post-exposure prophylaxis in humans bitten by rabies transmitting animals and losses to animals (ALE).

## 5 Conclusions

The higher engagement of the community in reporting dog bite cases during the one health intervention led to a higher calculation of the rabies burden in the animal and human health sectors in Ketapang District compared to the period before the one health intervention. The zDALY value after the one health implementation was also higher than the zDALY value observed without this approach. However, the zDALY rate with the one health approach is lower than the zDALY rate without the one health approach. This indicates that if preventive measures to curb disease’s spread to humans are not implemented, the disease burden will increase.

## Funding

This study was supported by the Ministry of Agriculture and Australia-Indonesia Health Security Partnership (AIHSP) with project collaboration registration number: 71465701 about strengthened health security in Indonesia.

## CRediT authorship contribution statement

**Cut Desna Aptriana:** Writing – review & editing, Writing – original draft, Project administration, Formal analysis, Data curation, Conceptualization. **Etih Sudarnika:** Writing – review & editing, Writing – original draft, Supervision, Formal analysis, Data curation, Conceptualization. **Chaerul Basri:** Writing – review & editing, Writing – original draft, Supervision, Data curation, Conceptualization. **Dikky Indrawan:** Writing – review & editing, Writing – original draft. **Joko Daryono:** Resources, Project administration, Funding acquisition. **Pebi Purwo Suseno:** Funding acquisition, Data curation.

## Declaration of competing interest

The authors declare that they have no known competing financial interests or personal relationships that could have appeared to influence the work reported in this paper.

## Data Availability

Data will be made available on request.
